# Overview of Solar Steam Devices from Materials and Structures

**DOI:** 10.3390/polym15122742

**Published:** 2023-06-19

**Authors:** Chang Liu, Zhenhao Yin, Yue Hou, Chengri Yin, Zhenxing Yin

**Affiliations:** 1 National Demonstration Centre for Experimental Chemistry Education, Department of Chemistry, Yanbian University, Yanji 133002, China; liuchang980726@163.com (C.L.); houyue178@163.com (Y.H.); 2 Department of Environmental Science, Yanbian University, Yanji 133002, China; yzh2015@ybu.edu.cn

**Keywords:** seawater desalination, interface solar evaporation, photothermal conversion, energy efficiency

## Abstract

The global shortage of freshwater supply has become an imminent problem. The high energy consumption of traditional desalination technology cannot meet the demand for sustainable energy development. Therefore, exploring new energy sources to obtain pure water has become one of the effective ways to solve the freshwater resource crisis. In recent years, solar steam technology which utilizes solar energy as the sole input source for photothermal conversion has shown to be sustainable, low-cost, and environmentally friendly, providing a viable low-carbon solution for freshwater supply. This review summarizes the latest developments in solar steam generators. The working principle of steam technology and the types of heating systems are described. The photothermal conversion mechanisms of different materials are illustrated. Emphasis is placed on describing strategies to optimize light absorption and improve steam efficiency from material properties to structural design. Finally, challenges in the development of solar steam devices are pointed out, aiming to provide new ideas for the development of solar steam devices and alleviate the shortage of freshwater resources.

## 1. Background and Significance

As the source of life for all things, water resources are vital to human survival and development. In recent years, water consumption has increased because of the high population growth and the industrialization of society. This has inevitably brought about an urgent problem of freshwater shortage [1,2,3]. The challenge of the global freshwater shortage has driven the development of various water purification technologies, among which desalination technology has shown potential for the application of water purification. It is considered one of the effective methods for solving the global freshwater resource crisis [4,5].

Desalination technologies commonly employed are divided into membrane and thermal methods [6,7,8]. As one of the main technologies, the membrane method includes reverse osmosis and electrodialysis. Reverse osmosis technology requires seawater pretreatment, where suspended solids and most harmful substances are blocked under the control of a semi-permeable membrane [9,10]. Then, the water source is pressurized by a high-pressure pump, which consumes mechanical energy to force fresh water through, thus retaining the salt. In recent years, the engineering costs and operating costs of reverse osmosis desalination technology have been continuously reduced. Despite significant advances in reverse osmosis membrane materials and technology, reverse osmosis membranes are less resistant to contamination and prone to fouling. Excessive insoluble residues can reduce membrane permeability to solutes and decrease membrane life [11,12]. Electrodialysis is a method of freshwater production using ion exchange membranes with direct current as the driving force [13]. This method is simple to operate, but it also requires raw water pretreatment. The energy consumption of this method is large when applied to seawater desalination, so it is basically not employed in large-scale seawater desalination projects. However, it is economical and feasible to desalinate 1000–3000 mg/L of seawater into 500 mg/L of drinkable water. Another desalination technology, the thermal method, with a high purity of produced water, is one of the existing mainstream technologies [14,15]. The conventional thermal distillation method requires heat energy generated from fossil fuel combustion to treat seawater, which is desalinated by heating to produce steam for condensation. The dependence on fossil fuels makes it vulnerable to factors such as the natural environment, energy prices, and process requirements [16,17,18]. With high investment costs and energy consumption, as well as the generation of environmentally harmful by-products, thermal distillation is not feasible in several developing countries, which cannot represent a long-term sustainable solution to the challenge of freshwater shortage. There is no doubt that the obtaining of clean fresh water is tightly linked to the consumption of energy. From the thermodynamics, the separation of water and salt ions is constrained by energy. In the era of energy sustainability, if consumed energy is available in low-carbon, abundant, and easy-to-capture ways, the burden of the whole energy system can be relieved effectively. For these reasons, solar-powered desalination is attracting attention as an effective green strategy. Solar energy, an eco-friendly renewable energy source with abundant reserves, is widely used in photocatalysis [19,20,21], photovoltaics [22,23], and photothermal conversion [24,25], which is one of the best energy options. The introduction of solar energy into seawater desalination not only promotes seawater evaporation but also effectively alleviates the energy crisis [26,27]. Therefore, developing steam devices driven by solar energy could provide a green and feasible solution for seawater desalination. It provides great potential for development in economically backward areas.

Various solar vapor devices have been recently developed for freshwater harvesting. Nevertheless, there are relatively few review studies on photothermal conversion materials and the overall structure of these devices. This paper systematically discusses the basic working principle of solar steam devices and the type of heating system. Recent research advances in materials and structures are described, as well as current challenges facing solar steam devices. Moreover, future research directions and improvement suggestions are presented to provide meaningful guidance for the research of solar steam technology.

## 2. Solar Steam Device

### 2.1. Working Principles of Solar Steam Technology

According to the energy transfer process, the operation of a solar steam device consists of three steps: light-to-heat conversion, heat-to-steam conversion, and steam condensation [28]. Photothermal materials interact with sunlight in the presence of the sun [29,30]. The light excites the particles in these materials and converts energy into heat. A portion of the heat is lost through thermal radiation at the solid-liquid interface, thermal convection, and heat conduction processes from the photothermal layer to the water [31]. Notably, most of the heat generated by the photothermal conversion is used to heat the water, which leads to a break in the ordered arrangement of water molecules. During this process, water molecules escape from the surface of the liquid into the air, generating steam [32,33]. Ultimately, the fresh water produced through condensate collection shows extremely low salinity, which is fully compliant with the drinking water standards set by the World Health Organization (WHO).

### 2.2. Types of Heating Systems and Solar Steam Generators

**Figure 1:**
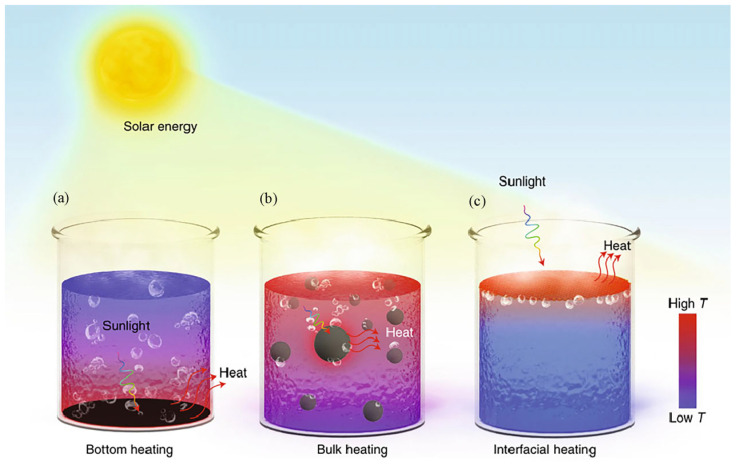
Solar-driven evaporation through various forms of solar heating. (**a**) Bottom heating model (**b**) Bulk heating model. (**c**) Interfacial heating model, reprinted with permission from [34], 2023, Springer Nature.

Depending on the location of the photothermal material, the solar steam systems can be divided into three different heating methods: bottom heating, bulk heating, and interfacial heating, as shown in Figure 1. Bottom heating is a photothermal conversion process that fixes active materials at the bottom of a water body. This method requires heating most of the water body (Figure 1a) [34]. Due to the long distance from the bottom to the surface of the water body, there is a heat transfer loss in addition to the light loss caused by water reflection. The generation of these losses reduces the energy use efficiency of the evaporator. Figure 1b represents the bulk heating with plasma nanoparticles as the photothermal material uniformly dispersed throughout the water body [35]. Since nanoparticles have excellent light absorption properties, when exposed to sunlight, the temperature rises rapidly, causing the nanoparticles to become encapsulated in water vapor [36]. Under continuous light exposure, the vapor layer propels the nanoparticles to float on the surface of the water. However, bulk heating can only improve energy efficiency to a certain extent [37]. Preventing the aggregation of nanoparticles to make them uniformly dispersed under long-term sunlight exposure becomes a challenge. Obviously, both of the above methods require heating of the entire water body, which has the problem of low photothermal conversion efficiency, leading to limited practical applications. To effectively reduce heat transfer loss, the interfacial heating method has been proposed in recent years (Figure 1c) [38,39]. Interfacial heating means a photothermal material is placed between the air and water interface so that the light is wholly focused on a thin layer of water on the evaporating surface. The heat obtained from the local heating is employed to heat the water surface to produce steam. This type of heating reduces the heat transfer to the water body and minimizes heat loss [40,41]. Therefore, applying solar-driven interfacial steam technology can improve the thermal localization of the phase change interface and achieve efficient water evaporation.

## 3. Photothermal Materials

Photothermal materials are the core components of solar steam devices [42,43]. The selection of photothermal materials with high solar absorption capacity is the basis for establishing an efficient solar evaporation system. Photothermal materials are mainly responsible for receiving sunlight to obtain energy, which is converted into heat energy through a light absorption mechanism [44]. The heat is then dispersed into the surrounding water body to promote rapid evaporation [41]. Since the heat to produce water vapor is mainly provided by solar energy conversion, the nature of the photothermal material directly determines the performance of the vapor generation system. Therefore, low emissivity, broad-spectrum light absorption, and efficient photothermal conversion capabilities are essential for photothermal materials [45,46]. In addition, to meet the requirements of practical applications, photothermal materials should also have good to excellent chemical stability [47].

Currently, photothermal materials can be classified into carbon, plasma metals, conjugated polymers, and inorganic semiconductor materials based on their chemical composition [48,49]. It is noted that each category has its own unique photothermal conversion mechanism [50]. In order to adapt to different natural environments and device structures, it is crucial to select a suitable material to improve the photothermal conversion efficiency of the device [51].

### 3.1. Carbon-Based Material

Among all the candidates, carbon materials exhibit significant advantages, which include excellent light absorption, good structural tunability, cheap cost, and high specific surface area to meet the high requirements of solar steam devices [52,53]. Furthermore, carbon materials are abundant and generally include graphite-based (graphite, graphene, graphene oxide, and reduced graphene oxide), carbon nanotubes (CNTs), amorphous carbon (carbon black and activated carbon), and carbon composites [54,55,56]. Because of the black surface of carbon materials, they have high solar absorption properties, which is the main reason for their wide range of visible light absorption [57]. In light absorption, carbon materials undergo photothermal conversion through intermolecular vibrations. Under the sun’s irradiation, most of the photons in the light waves are absorbed by the electrons on the surface of the carbon material. The electrons that gain energy undergo energy level jumps, thus converting solar energy into thermal energy, which is released as heat [58,59]. Additionally, carbon materials could be coupled with other photothermal materials to form composite materials with excellent properties. The synergistic effect of multiple materials can effectively improve light absorption and desalination performance.

**Figure 2:**
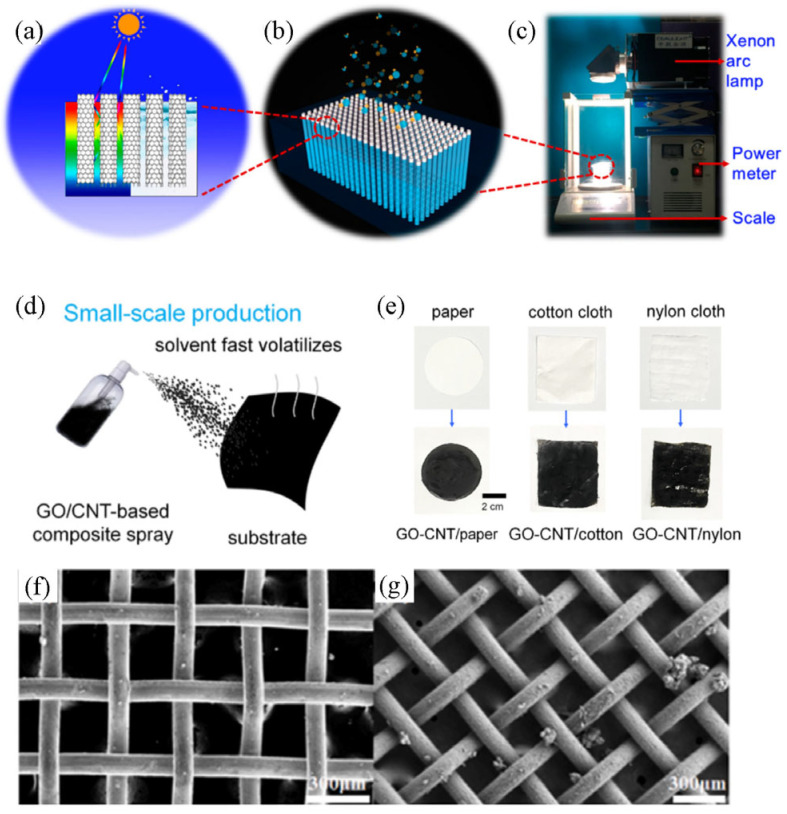
Carbon as photothermal materials for solar evaporators. (**a**) Structural diagram of the vertically aligned carbon nanotube array. (**b**) Microscopic schematic diagram of the carbon nanotube array. (**c**) Solar vapor generation device composed of carbon nanotubes, reprinted with permission from [60], 2017, American Chemical Society. (**d**,**e**) GO/CNT were sprayed onto the thin film evaporator as a photothermal material, reprinted with permission from [40], 2022, American Chemical Society. (**f**,**g**) SEM images of the CNTs deposited on a porous nickel grid are shown, forming a rough surface, reprinted with permission from [62], 2022, Elsevier.

CNTs are one of the typical photothermal materials, which structurally feature large specific surface areas that help to enhance the local temperature of the surface, giving it good light absorption properties. Yin et al. [60] designed vertically aligned CNTs as the photothermal layer to achieve local heating by capturing an array of sunlight, which effectively improved the light absorption rate during device operation (as shown in Figure 2a–c). Hence, the evaporation efficiency of this structure is ten times higher than that of ordinary pure water evaporation and its solar thermal conversion efficiency is 90%. Compared with conventional carbon-based materials, reduced graphene oxide (rGO) has high light absorption properties. Meanwhile, it exhibits excellent high-temperature tolerance under long-term solar irradiation, which is one of the ideal properties for photothermal materials. Liao et al. [61] prepared a thin-film spectrally selective absorber by adjusting the thickness and reduction degree of internal two-dimensional (2D) graphene. The device consists of an aluminum substrate and an rGO film. This transmitter relies on high-temperature resistance and an excellent heat transfer ability to achieve a net water content of 0.94 mg cm^−2^·s^−1^ under six simulated sunlight conditions. More importantly, the device exhibits a high-temperature tolerance of 96 h at 800 °C and a theoretical long-term stability of 25 years at 177 °C. In addition, carbon-based composites have shown excellent performance in the field of solar interface evaporation. Zhang et al. [40] prepared GO/CNT as a photothermal material for thin-film evaporators. As shown in Figure 2d,e, they combined the industrial production technique with the spray method to evenly coat the carbon material on the substrate, thereby obtaining the thin-film evaporator. The evaporation rate of the thin-film evaporator prepared under optimal conditions was 2.1 kg·m^−2^·h^−1^ and the evaporation efficiency was 90.1% under a single solar irradiation.

Except for the composite between carbon materials, high absorption properties could also be demonstrated by combining carbon with other materials. Li et al. [62] constructed carbon-based composites by combining porous nickel mesh and CNTs using a chemical vapor deposition technique and growing CNTs on porous nickel mesh (Figure 2f,g). Owing to the porous structure and rough surface of the material, incident light is reflected multiple times inside the channel. The device can efficiently utilize solar energy (94.3%) with an evaporation rate of 2.13 kg·m^−1^ under a single solar irradiation. Moreover, the corrosion resistance of nickel gives the device excellent and stable decontamination abilities, which can maintain a relatively stable performance during the evaporation cycle within 46 h.

Besides the typical carbon-based materials mentioned above, methods of biological carbon production have been developed. Many biomass materials with biodegradability, low cost, and natural capillary mechanism properties are suitable for photothermal evaporation. Zhu et al. [63] used low-cost and abundant white radish as the raw material, which was freeze-dried, and then carbonized. The carbonized white radish had a highly developed honeycomb structure with dense pores to enhance light capture, had good hydrophilicity and had interconnected channels that provided a strong capillary force for rapid water transport. The results showed that the water evaporation rate of charred white radish was 1.57 kg·m^−2^·h^−1^ under a single solar irradiation, achieving effective water purification. It is worth noting that the solar steam efficiency did not exhibit a significant decrease after 10 cycles. Zhou et al. [64] used cheap and durable charred tofu for the first time as a photothermal material for efficient solar steam power generation. Charred tofu has a rich porosity and is simple to manufacture. The device has fast water transfer performance, good hydrophilicity, high salt tolerance, and low thermal conductivity (0.093 W·m^−1^·k^−1^). The evaporation rate was 1.65 kg·m^−2^·h^−1^ under a single solar irradiation, which resulted in a photothermal conversion efficiency of 87.26%. Importantly, after 25 days of salt tolerance testing, although the material had crystals on the surface, it could be reused after soaking in water. Although natural biomass is easily recyclable and low in cost, it generally provides relatively low light absorption properties. Therefore, it usually requires additional nanomaterials or chemical treatments to provide surface coatings and material carbonization to improve performance.

Comprehensively, the photothermal conversion efficiency of the carbon-based material can be maintained after being irradiated with solar light several times. However, the absorption rate of carbon-based materials is limited by the light reflection of the surface. Improving the light absorption capacity of carbon-based materials is mainly achieved with high absorption intensity and low reflectance [65]. The most effective strategy is to design nanostructures with different geometries, such as porous, arrays, and multistage nanostructures. By utilizing these structures, the effective propagation path of light can be extended, thereby increasing light reflection and scattering, enhancing light absorption and reducing the dependence on incident angle [66,67,68,69,70]. Nevertheless, carbon materials, including carbon nanotubes, activated carbon, and rGO, exhibit natural hydrophobicity, which inhibits the transport of liquids [71,72,73]. Therefore, complex hydrophilic modification treatments are generally required for applications. Additionally, the high thermal conductivity of carbon materials rapidly releases the heat obtained from conversion into the surrounding environment, increasing the heat transfer losses, which further reduces the evaporation rate of water. The poor anti-fouling ability and low antimicrobial performance make carbon-based material susceptible to microbial adhesion, which is also an important factor affecting the performance of the device.

### 3.2. Plasma Metal

**Figure 3:**
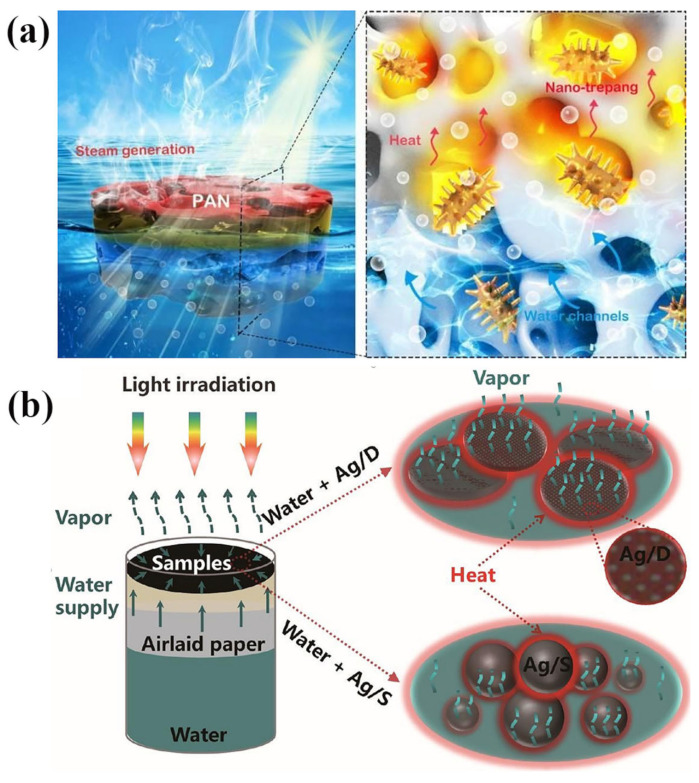
Photothermal material with plasma as a solar evaporator. (**a**) Schematic diagram of the device vapor generation with a novel sea cucumber-like nanostructure as the photothermal layer; reprinted with permission from [75], 2020, Royal Society of Chemistry. (**b**) Schematic diagram of the solar steam generating device composed of Ag and diatomite earth; reprinted with permission from [79], 2017, Royal Society of Chemistry.

Local surface plasmon resonance (LSPR) is an optical phenomenon unique to nanometals, which can effectively capture sunlight through this effect [74]. The reason for this phenomenon is the strong absorption of surface plasma by solar radiation. When electrons in plasma metal are excited by incident light, the received energy is converted into thermal energy through scattering, rapidly increasing the surface temperature of metal nanoparticles and exhibiting a rapid light response [75,76]. Gold, silver, germanium, and other metal nanoparticles with LSPR can locate heat in the nanoscale area. By enhancing the light–matter interaction between small volumes, it is beneficial to reduce the loss of heat and light [77]. In addition, plasma metal materials have good antibacterial properties, demonstrating broad application prospects in seawater desalination. Under sunlight irradiation, metal nanoparticles with LSPR effects typically exhibit absorption and scattering of the incident light, which drives local heating and increases the possibility of efficient hot vapor generation by the device. Huang et al. [75] developed a novel sea cucumber-like gold nanostructured plasmon absorber as shown in Figure 3a, which exhibited good light absorption (92.9%) by adjusting parameters, such as nanostructure and morphology. The absorber stability data of 2.7 kg·m^−2^·h^−1^ could be obtained under the irradiation of one sun (AM 1.5 G). Compared with gold, silver has the advantage of low cost among precious metals and can replace gold nanoparticles. Wang et al. [78] assembled a solar vapor generation device using spherical silver nanoparticles as the light absorber. Under solar irradiation, the device exhibited a thermal conversion rate of 82.45% and an evaporation rate of 16.80 g·m^−2^·min^−1^ (AM1.5 G). Fang et al. [79] constructed an efficient solar vapor generator by uniformly depositing silver nanoparticles on inexpensive diatomaceous earth and combining them with filter paper and polystyrene foam (Figure 3b). The evaporation rate and efficiency were 1.39 kg·m^−2^·h^−1^ and 92.2%, respectively. In addition to being directly used in photothermal materials, metal nanoparticles can also be combined with polymers to improve device performance further. Fan et al. [80] deposited silver nanoparticles (AgNPs) and polydopamine (PDA) on natural winter melon substrates by a simple immersion method. The synergistic photothermal conversion of the evaporator was enhanced mainly by the local SPR effect of AgNPs and the light absorption ability of PDA. The prepared evaporator reached 1.70 kg·m^−2^·h^−1^ with an efficiency of 83.21% under a single solar irradiation. In addition, excellent antibacterial properties (against *E. coli* and *S. aureus*) were demonstrated in practical applications. The device presented good durability during 10-cycled experiments, seawater desalination, dye water purification, and some extreme conditions.

Based on the LSPR effect, plasmonic metal nanoparticles effectively increase the evaporation rate of water, which provides a new strategy for maximizing the conversion with solar energy [76]. Commonly applied plasmas include Al, Ag, Ge, and composite metals. Although nanoparticles have excellent light absorption efficiency, the scarcity and high cost of precious metals limit large-scale utilization. Moreover, the instability and toxicity of plasma metal nanoparticles are also difficult problems that plague practical applications. When metal particles are subjected to multiple solar radiations, the structure tends to be damaged, which leads to a change in absorption wavelengths, resulting in a large reduction in photothermal conversion efficiency. More importantly, most plasma metals have the defect of a narrow absorption range, so it is necessary to introduce porous nano/micron structures or carefully arranged distributions to induce plasma coupling to extend the light absorption range and improve light absorption performance. In addition, the dispersion of low-stability of nanoparticles needs to be improved.

### 3.3. Conjugated Polymer

Conjugated polymers are compounds consisting of alternating single- and double-bonded conjugated systems that are easy to prepare and have tunable pore structures, such as polypyrrole, polyaniline, and polythiophene [56]. In addition, the photothermal conversion process relies on the delocalization of π-electrons, which gives them a strong solar energy absorption capacity. The conversion of incident photons into thermal energy through non-radiative relaxation and lattice vibrations in the conjugated structure enables effective solar light harvesting [81]. In addition, the polymer can combine two or more photothermal agents in addition to good adhesion to the support structure when used as a photothermal material, demonstrating remarkable compatibility.

However, the narrow absorption bandwidth of conjugated polymers, influenced by their relative molecular weights and structures, reduces the energy conversion efficiency to full-spectrum sunlight. A series of modification strategies, such as molecular structure design and doping (oxidative doping and plasmonic acid doping), have been developed to address these issues [82,83]. The spectral range of the modified conjugated polymers can be broadened into the infrared region, thus maximizing light absorption.

**Figure 4:**
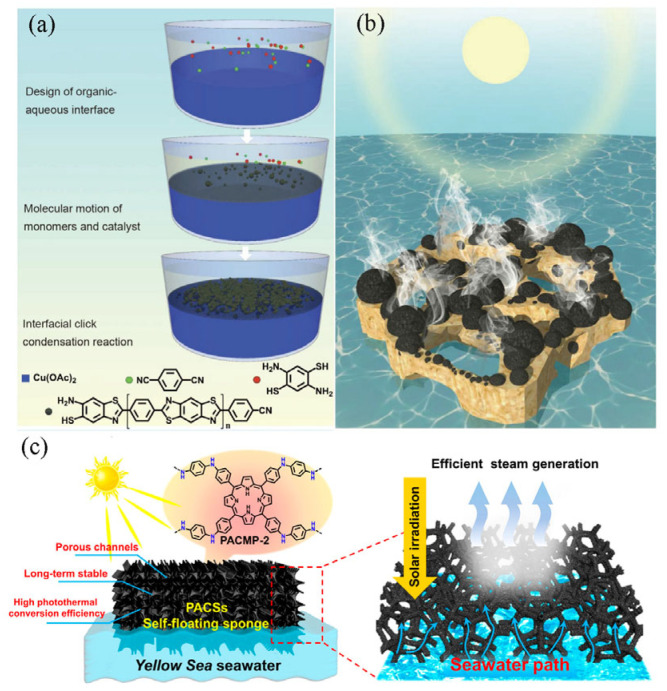
Solar vapor generation device with conjugated polymers as photothermal materials. (**a**) Schematic diagram of the polymerization process of conjugated polyphenylene diazole microspheres. (**b**) Structure of the evaporation device with microspheres combined with polyvinyl alcohol hydrogel; reprinted with permission from [81], 2022, Springer Nature. (**c**) Solar energy evaporator composed of conjugated microporous polymer; reprinted with permission from [87], 2022, American Chemical Society.

Wu et al. [84] developed a new polypyrrole hydrogel with butane tetracarboxylic acid (BTA) as a dopant for solar vapor generation. BTA promoted the formation of a strongly hydrophilic polypyrrole hydrogel, and a high evaporation rate (1.90 kg·m^−2^·h^−1^) and energy conversion efficiency (89%) were obtained under one sunlight. In addition, the evaporation rate stays between 1.78 and 1.82 kg·m^−2^·h^−1^ after 50 cycles, demonstrating excellent stability. Zhao et al. [81] synthesized conjugated polyphenylene diazole microspheres with a narrow-forbidden bandwidth of 0.274 eV and a high solar absorption of 94.0% (Figure 4a,b). The rough surface of the heterogeneously sized microspheres allows for enhanced solar energy collection through multiple light scattering and diffuse reflection. The efficiency of a single solar evaporation device with hydrophilic microspheres combined with polyvinyl alcohol hydrogel was 2.96 kg·m^−2^·h^−1^. Alternatively, some conjugated microporous polymers (CMPs) have strong light absorption ability in the near-infrared region, have high porosity, and have a large specific surface area, providing abundant fluid channel mass transfer for heat exchange and making them ideal solar steam materials. Mu et al. [85] developed a new solid aerogel based on conjugated microporous polymer nanotubes via Sonogashira—Hagihara cross-coupling reaction with 1,3,5-triacetylbenzene, 1,4-dibromobenzene, and 4,4-dibromobiphenyl as raw materials under the catalysis of Pd (0)/CuI. This material has excellent mechanical strength and high porosity (94%) with a vapor efficiency of 81% at 1 kW·m^−2^ of light intensity. A heterocyclic CMP aerogel solar receiver was reported by Zhu et al. [86]. The aerogel was prepared from 2-amino-3,5-dibromopyridine and 1,3,5-triethynylbenzene with polypyrrole, and then uniformly sprayed on the surface of the aerogel to obtain a solar vapor generator. The device exhibits high porosity and excellent light absorption performance, with a vapor generation efficiency of up to 88%. Except for the use of a single polymer as the photothermal material, the integrated action of two or more materials makes the conjugated polymer composites potentially great for photothermal conversion. Shi et al. [87] synthesized a novel porphyrin/aniline-based conjugated microporous polymer (PACMP), which was immersed in a polyurethane sponge substrate to complete the evaporator structure, as shown in Figure 4c. The evaporation rate of seawater under standard solar radiation (1 kW·m^−2^) was 1.31 kg·m^−2^·h^−1^, and the solar thermal conversion efficiency was 86.3%.

Environmentally friendly, lightweight and flexible conjugated polymers have emerged as promising photothermal materials for solar evaporation. Moreover, some conjugated polymers with interconnected porous network structures also facilitate water transport, offering a possibility of substrate selection. However, conjugated polymers have disadvantages, such as poor photothermal stability, short service life, and easy aging. Meanwhile, due to the characteristics of alternating single and double bonds in the molecule, conjugated polymers tend to twist, which causes molecular agglomeration and entanglement. This molecular property makes it weakly resistant to photobleaching, leading to easy decomposition after solar irradiation, which directly hinders its large-scale application. Moreover, polymers of a given molecular weight and structure only possess a single energy band conformation, resulting in a narrow absorption bandwidth and, hence, the low energy conversion efficiency of polymers for full-spectrum sunlight. As an effective strategy, conjugated polymers are compounded with other photothermal materials to compensate for the lack of a single molecule.

### 3.4. Inorganic Semiconductor

**Figure 5:**
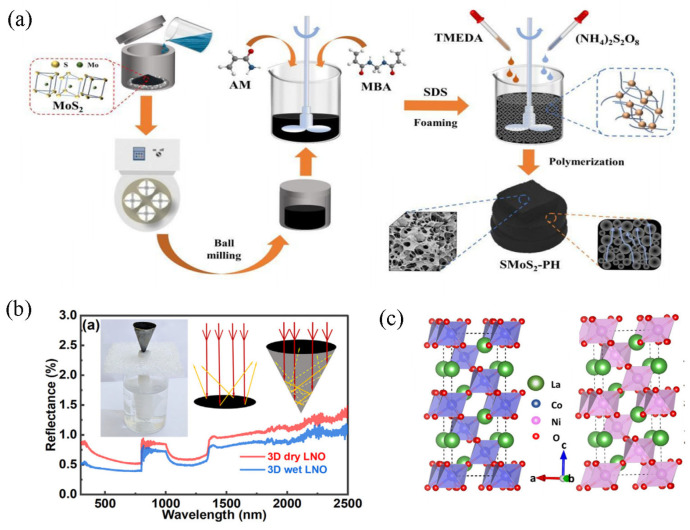
Water evaporation device using semiconductor material as a photothermal layer. (**a**) Preparation of porous hydrogel based on MoS_2;_ reprinted with permission from [92], 2022, John Wiley and Sons. (**b**) Conical 3D structure when using metal oxides as a light-absorbing layer. (**c**) Schematic diagram of the crystal structures of LCO and LNO; reprinted with permission from [94], 2022, Elsevier.

Inorganic semiconductor materials based on metal oxides and metal sulfides exhibit excellent light absorption properties, which have been demonstrated for the desalination of seawater. Normally, the electronic energy band structure of inorganic semiconductors is the main factor that determines their light absorption properties [88]. From a microscopic point of view, semiconductors have two main different photothermal conversion mechanisms. One is based on the SPR effect [89], whereby surface charge carriers undergo a migration process when a defective structure is present in the semiconductor material. This process generates the plasmon resonance effect, which eventually leads to broader light absorption [90]. The other one is the non-radiative relaxation caused by the intrinsic bandgap absorption of the semiconductor. When incident light is irradiated, photon absorption and semiconductor bandgap pairing excite molecules, generating electron-hole pairs. Electrons and holes enter the edges of the conduction and valence bands before recombining, converting radiated energy into heat. It can be seen that the bandgap energy of semiconductors directly determines their ability to absorb solar energy [91]. Traditional wide bandgap semiconductors typically only react to ultraviolet light, while narrow bandgap semiconductors tend to generate electron-hole pairs. Therefore, bandgap engineering is the key to improving the photothermal performance of semiconductors. Inorganic semiconductor materials (metal sulfides, metal oxides, etc.) with narrow band gap properties achieve efficient light energy utilization under sunlight. Liu et al. [92] used molybdenum disulfide (MoS_2_) as the active material loaded onto a hydrophilic polyacrylamide backbone to prepare the porous hydrogel for application in steam devices. The preparation process is shown in Figure 5a. The constructed solar evaporation devices have good mechanical stability and specific salt resistance. The evaporation rate is 3.29 kg·m^−2^·h^−1^, and the photothermal conversion efficiency of primary solar energy is 93.4%. Excitingly, the device can maintain highly stable photothermal performance on the surface of seawater for 15 days. In the design of photothermal materials, the method of combining hydrogels with functional materials also makes it possible for efficient solar steam devices. Irshad et al. [93] developed a polymeric hydrogel network prepared from MnO_2_ nanowires/chitosan (CS) for continuous solar energy-driven vapor generation. This hydrogel, which controls non-radiative relaxation phenomena to generate thermal energy, shows great potential with high light absorption (94%) under sunlight irradiation. In nature, transition metal oxides have the characteristics of large reserves and environmental stability, especially lanthanum-based composite oxides. Wani et al. [94] synthesized the metal oxides of LaNiO_3_ and LaCoO_3_ as photothermal materials for solar evaporators and achieved evaporation rates of 1.45 kg·m^−2^·h^−1^ and 1.38 kg·m^−2^·h^−1^, respectively. Furthermore, it is worth mentioning that the team has additionally designed a conical three-dimensional (3D) structure to achieve efficient evaporation (Figure 5b,c). On this structure, the maximum evaporation was achieved when LaNiO_3_ was the photothermal layer, which succeeded in achieving an evaporation rate of 2.3 kg·m^−2^·h^−1^ (83% of photothermal efficiency).

Inorganic semiconductor materials (metal sulfides, metal oxides, etc.) with narrow band gap properties achieve efficient light energy utilization under sunlight. Inorganic semiconductor materials with photothermal properties show extraordinary research potential for desalination because of their controllable structure and high chemical stability. Notably, the wide band gap of semiconductors poses a challenge for a wide range of applications [95]. In general, semiconductors with a narrow band gaps are able to induce carrier flow. The high carrier concentration of the material could lead to the LSPR effect, which increases the absorption range of light and enables the effective use of light energy. There are many ways to decrease effectively the bandgap of semiconductors, one of which is chemical doping. Impurities are introduced into the crystal structure of the material to tune the energy band structure. By changing the position of the valence and conduction bands, the absorption range of light is thereby expanded. Except for doping, the energy band gap could be narrowed by changing the composition of the semiconductor, which varies the concentration of free carriers in different material compositions. When the absorbed energy is higher than its band gap, the concentration of free carriers is higher, with the material tending to achieve greater light-trapping capabilities. Additionally, the low recyclability of semiconductor materials causes inevitable environmental pollution, so it becomes an issue to be considered for practical applications.

Table 1 shows the comparison of water desalination parameters of materials with different photothermal mechanisms under one solar irradiation. In general, capturing sunlight is crucial for effective photothermal vapor generation. On the one hand, in terms of performance, suitable photothermal materials should have excellent light absorption properties and high chemical stability. The operation of the solar desalination device relies on efficient light energy utilization. Therefore, the light absorption capacity directly determines the efficiency of the device. Furthermore, solar steam generators that work outdoors inevitably face extreme weather challenges in the natural environment. A high-performance device needs to ensure stable efficiency even under long-cycle operation. Hence, it is essential to develop new photothermal materials with high chemical stability. On the other hand, the structures for solar thermal conversion materials should be porous to facilitate multiple light absorptions, conducive to direct water-gas transformation, which enables efficient interfacial evaporation.

## 4. Design of Device Structure

In recent years, interfacial solar thermal-vapor conversion efficiency has increased to about 90%, thanks to the rapid development of new solar thermal conversion materials and the effective regulation of the device structure. In addition to relying on photothermal materials to adequately collect sunlight and efficiently convert it into heat, the structural design of the equipment can further reduce heat loss [45]. The current common structures of solar interface evaporators include double-layer, 3D, and bionic structures.

### 4.1. Double-Layer Structure

**Figure 6:**
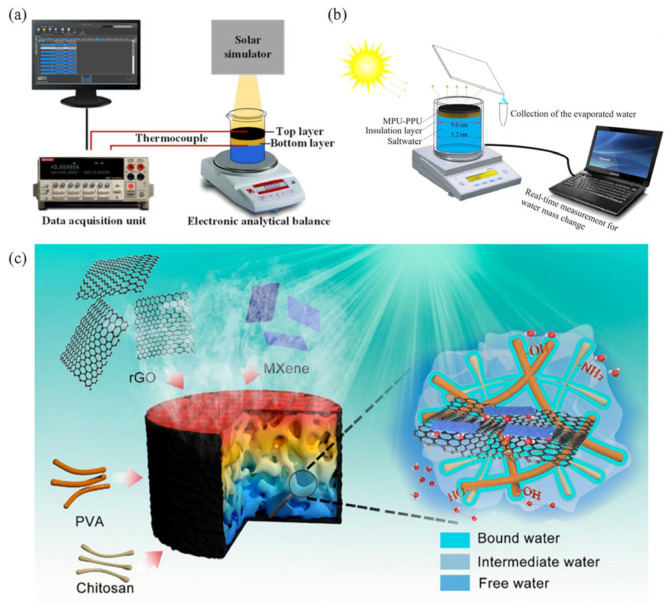
A schematic diagram of the structure of a two-layer solar vaporization power generator. (**a**) Device with carbon material as the absorber layer and wood as the insulating layer; reprinted with permission from [100], 2018, John Wiley and Sons. (**b**) Device where the insulating and absorbing layers are made of PU materials loaded with different active substances; reprinted with permission from [101], 2020, Elsevier. (**c**) Schematic diagram of a hybrid hydrogel with transition metal carbides embedded in a 2D nanostructure; reprinted with permission from [102], 2021, American Chemical Society.

A typical double-layer interface solar desalination system usually consists of the light-absorbing layer and the thermal-insulation layer. The role of the light-absorbing layer is photothermal conversion, heating the seawater pumped by capillary action. The thermal-insulation layer usually consists of a porous absorbing material with low thermal conductivity. The advantage of materials with high porosity is the ability to reduce heat loss in transport, confining the heat to the surface, which enables localized heating [96,97]. Moreover, the thermal-insulation layer should not only prevent heat loss and transport of moisture but also act as the support layer for the photothermal material, which keeps the evaporator stable in the natural environment [98,99]. On the basis of the double-layer structure strategy, various 2D planar evaporation devices have been successfully developed recently. Notably, insulation materials, such as cellulose foam, polystyrene foam, and natural wood, have been explored. As shown in Figure 6a, Luo et al. [100] designed a two-layer solar evaporator using low-cost carbon particles as the absorber layer and boxwood as the support layer. They can provide good water transport and thermal management and improve the stability of the device due to the excellent light absorption properties of the carbon-based material and the excellent local heating management advantages of the wood material. The device provides 65% evaporation efficiency at one solar lighting power. Besides wood, a sponge, with a good capillary effect as an insulation layer, is also a promising strategy. Wang et al. [101] proposed a solar evaporator designed with a double-layer structure (Figure 6b). The light-absorbing layer consists of nano-metal MoS_2_ immobilized on a 3D porous polyurethane (PU) sponge. The thermal-insulation layer consists of a PU sponge with hydrophilic PDA coating for fluid transfer. The evaporation efficiency is 86% at a low light level of 1.0 kW·m^−2^ and over 90% at a high solar light level of 1.5–2.5 kW·m^−2^. Lu et al. [102] constructed a hybrid hydrogel with embedded 2D nanostructures and the surface of which is a concave pyramidal pattern. Multilayered 2D titanium carbide (Ti_3_C_2_T_x_ MXene) and rGO nanosheets were simultaneously penetrated into a polymer network of polyvinyl alcohol and CS. The advantage of this design is that it regulates the flow rate of water, enhances light absorption near the surface, and thus forms an efficient device that improves the evaporation rate from 3.38 to 3.62 kg·m^−2^·h^−1^ (Figure 6c).

The solar evaporative photothermal conversion material dispersed into the overall water floats to the air-water interface, enabling a direct transition from water to gas. It means that the photothermal conversion system shifts from a low-dimensional to a floating 2D interface [103,104]. By controlling the energy on the surface of the water body with a double-layered structure, it provides new research ideas and directions for solar desalination technology as well as promotes the development of solar desalination systems. Nevertheless, the upper part of the 2D interfacial solar evaporation structure is an open type of space which is connected to the environment directly. Since the diffuse reflection of the incident light and the external thermal radiation are the main energy losses, the efficiency of interfacial solar evaporation in the 2D plane has reached its upper limit. Based on the limited capability and functionality of producing clean water, the 2D structures still face many challenges in practical applications.

### 4.2. Three-Dimensional Structure

**Figure 7:**
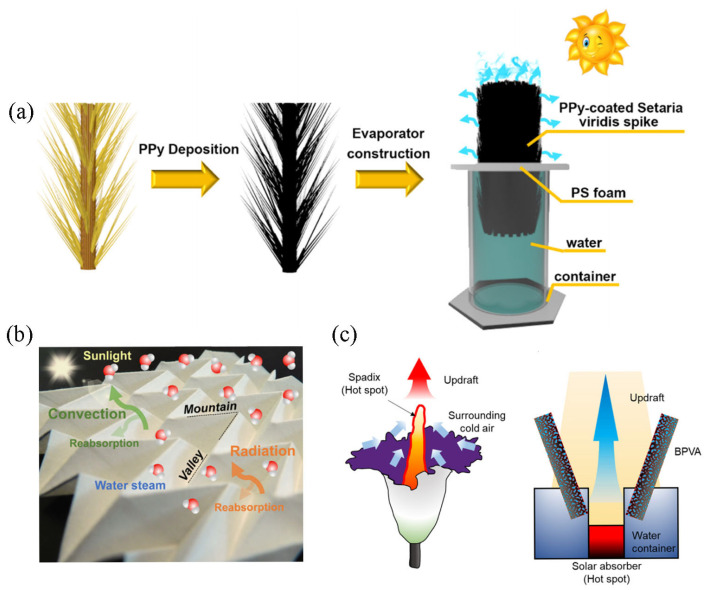
Schematic diagram of the different 3D structures. (**a**) A 3D evaporation device based on *S. viridis*; reprinted with permission from [109], 2021, American Chemical Society. (**b**) An origami-type solar evaporator capable of free folding and unfolding; reprinted with permission from [110], 2018, American Chemical Society. (**c**) A funnel-shaped device with the structural design of a convection flower and a solar chimney as a reference; reprinted with permission from [111], 2021, American Chemical Society.

Unlike typical 2D planar structures, evaporators with 3D structures have more significant evaporation efficiency for the following reasons [105,106]. First, the evaporation rate is closely related to the surface area exposed to irradiation. The unique sides of the 3D system serve as additional light-receiving surfaces that provide sufficient space for vapor release. Secondly, the internal porous design of the 3D structure can effectively capture sunlight. In the interior of the 3D material, sunlight can be reflected and refracted several times to improve the utilization of solar energy. Finally, the 3D structure could make the light absorption area larger than that under irradiation, thereby enhancing the use of light and maximizing the recovery of heat loss. Additionally, the 3D structure cleverly utilizes air convection, which removes water vapor accumulated on the surface of the photothermal material to promote continuous evaporation [107,108]. Consequently, evaporators based on 3D structures maximize the interfacial evaporation rate of solar energy under the synergistic effect of radiation with convection. Currently, the efficiency of 3D solar vaporizers is usually slightly higher than 2D devices. As shown in Figure 7a, Xie et al. [109] prepared solar evaporators using the stigma of Setaria viridis (*S. viridis*) as the substrate, which was covered with polypyridine (PPy). The green *S. viridis* stigma has a specific surface area that constitutes the 3D structure. This solar evaporator achieves an evaporation rate of up to 3.72 kg·m^−2^·h^−1^ owing to the increased evaporation area and the open evaporation structure. Hong et al. [110] have designed a deployable 3D origami-type solar vaporizer by utilizing a nanocomposite composed of GO and CNTs as the photothermal material, which highly captures solar energy via the folds created by folding and unfolding (Figure 7b). The device is stable due to periodic folding and produces nearly 100% solar energy efficiency in a single solar irradiation. As in Figure 7c, Kim et al. [111] reported a 3D solar vaporization device that combines the structural design of a convective flower (*Amorphophallus titanum*) and a solar chimney, consisting mainly of a funnel-shaped carbon-coated polyethylene hydrocarbon foam and a dry solar absorber zone. A central hot spot in the absorber zone generates a solar-induced updraft to remove excess water vapor and accelerate evaporation efficiency. The carbon-coated polyvinyl alcohol (PVA) foam with good light-absorbing properties can supply water continuously through the entire duty cycle. After subtracting the evaporation rate under dark conditions, it achieves a solar thermal efficiency of 95.9%.

Usually, 3D evaporation systems can effectively reduce heat loss and light reflection or increase evaporation area and absorption, thus exhibiting excellent evaporation performance [106]. When designing 3D structures, there are many strategies to improve the interface evaporation rate besides compensating for the incidence angle effect. One of the strategies is to reduce diffuse reflections through excellent light management, and thus enabling multi-directional light absorption. Furthermore, optimizing thermal management to suppress heat loss is also an effective strategy for improving heat utilization. Appropriate expansion of the evaporation area to increase the effective light absorption surface could also enhance the net water rate [112]. Eventually, the large area within the system is used to obtain energy from the environment. The variation from a 2D to a 3D interfacial evaporation system is not only a volume change, but benefits from the increase in light absorption surface, which effectively increases the evaporation rate. However, the actual operation of the 3D evaporation system has yet to be explored because of the complexity and high cost of the current fabrication process.

### 4.3. Biomimetic Structure

Motivated by organisms in nature, researchers have designed a series of efficient solar evaporators by simulating their structure and growth mechanisms. These evaporators can cope with high-frequency environmental changes while possessing high evaporation efficiency, cycle stability, and desalination rate [113,114]. For example, plants mainly composed of cellulose have excellent hydrophilicity and low thermal conductivity, meeting the needs of water channels and thermal insulation materials. Secondly, the multi-dimensional ordered pores of the plant structure could significantly improve the light absorption of the material. This structural design not only suppresses the energy loss generated during the photothermal conversion process but also achieves multi-directional light absorption, which is very suitable for the field of efficient seawater desalination [115]. Therefore, preparing solar evaporation devices using plant bionics has become a hot field.

**Figure 8:**
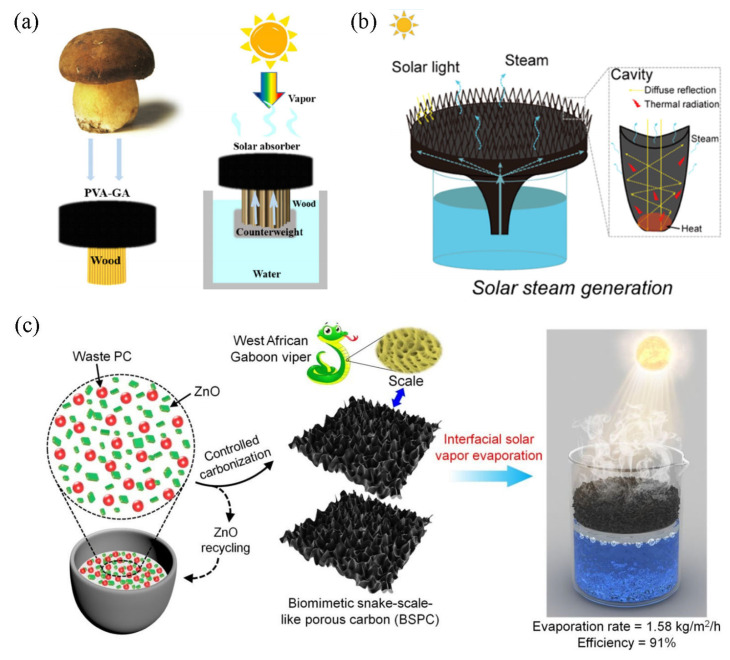
Schematic diagrams of various devices with bionic structures. (**a**) A solar evaporator inspired by the mushroom form in nature; reprinted with permission from [116], 2022, Elsevier. (**b**) Sunflower 3D structure inspired device for tracking sunlight; reprinted with permission from [117], 2020, American Chemical Society. (**c**) Solar evaporator designed to mimic animal scales; reprinted with permission from [118], 2020, Royal Society of Chemistry.

Wang et al. [116] have developed a novel and efficient biomimetic mushroom solar steam generator (BMSSG) inspired by the transpiration of trees, as shown in Figure 8a. The steam generator, which can float stably on water, uses wooden slats to mimic mushroom stems and polyvinyl alcohol-modified graphene aerogel to mimic mushroom caps with evaporation rates up to 1.67 kg·m^−2^·h^−1^. The length of the slats is longer than the immersion depth, making the air layer an insulating layer. Based on the evapotranspiration of natural trees, this design optimizes the evaporation rate by adjusting the aerogel’s composition and the slats’ length. Thus, using a simple integrated structure helps improve stability and reliability while reducing manufacturing costs. Inspired by the sun-facing properties of sunflower seed heads, Sun et al. [117] used the 3D structure of carbonized sunflower seed heads as an effective solar steam generator (Figure 8b). The main working mechanism is to raise the temperature of the disc-shaped flowers by tracking sunlight. The advantage is that many sunflower seed by-products are not fully used as bio-waste but are stored in large quantities. The disc-shaped sunflower contains abundant spongy tissues that exhibit strong light absorption ability depending on its inherent 3D structure. The vascular bundles formed during growth can also transport water/air. Therefore, sunflower heads are one of the natural biological candidates suitable for solar steam power generation. The laminar porous structure of the carbonized sunflower seed head combined with the 3D macroscopic shape effectively improves the evaporation performance of the system. The evaporation rate was 1.51 kg·m^−2^·h^−1^ at 1 kW·m^−2^ and the evaporation efficiency was 100.4%, exceeding the 2D material’s theoretical limit.

The microstructure of animals is more delicate and complex than that of plants, for which some researchers have also turned their attention to animal bionics in recent years. Based on the unique hierarchical structure of Gabonese viper scales, Liu et al. [118] utilized ZnO-catalyzed porous carbon (PC) waste “controlled carbonization” to prepare low-cost serpentine PC, thereby developing an efficient solar energy absorption and water evaporation system (Figure 8c). ZnO catalyzes the decarboxylation of PC, producing microporous, mesoporous, and macroporous structures. Due to its 3D interconnected hierarchical nanopores and oxygen-rich functional groups, it exhibits strong hydrophilicity. The prepared serpentine-grade PC has a high solar energy absorption rate (~95%), low thermal conductivity (0.086 W·m^−1^·K^−1^), high solar thermal conversion efficiency, and fast water transfer ability. At 1 kW·m^−2^, the evaporation rate is 1.58 kg·m^−2^·h^−1^ and the conversion efficiency is 91%.

Nature is a master at building all kinds of delicate structures, so plants and animals have inspired researchers. The emergence of bionic design breaks the typical structure inherent in solar interface evaporation devices. Compared with other structures, biomimetic structures are more harmonious with the natural environment. However, the design of the bionic structure is very cumbersome, thereby greatly increasing the operating cost. In addition, as the emerging special structure, its stability and photothermal conversion efficiency still need to be improved, this is currently an urgent problem to be solved.

### 4.4. Other Structures

**Figure 9:**
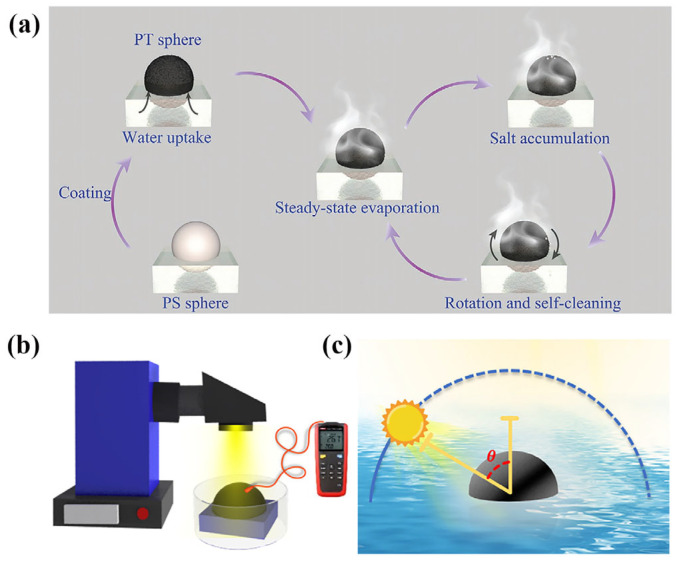
Schematic diagram of the structure of other types of devices. (**a**) Spherical self-rotating double evaporation zone (high and low-temperature evaporation zones) evaporator; reprinted with permission from [119], 2021, John Wiley and Sons. (**b**) Structure of a photothermally formed evaporator with inverted bowl-shaped graphene aerogel. (**c**) Schematic diagram of the incidence angle of sunlight; reprinted with permission from [120], 2022, John Wiley and Sons.

In addition to the above-mentioned typical structures, it is crucial to design and develop new structures to achieve strong salt resistance of the device. The reason is that in the case of continuous evaporation, the concentration of salt ions on the evaporation surface is greater than that in the water body. When the concentration of salt ions reaches saturation, the salt would easily crystalize and precipitate, which increases the reflection of sunlight, resulting in weak light absorption. Furthermore, the accumulation of salt can block the steam discharge orifice and obstruct the water delivery, resulting in a lower evaporation efficiency of the equipment. Figure 9a shows that Wu et al. [119] developed a spherical self-rotating double evaporation zone (high and low-temperature evaporation zones) evaporator. The double evaporation zone is sensitive to weight imbalances (<15 mg) under continuous solar irradiation, which causes a rotation triggered by salt accumulation at the top of the spherical evaporator. This double evaporation zone could respond quickly to salt accumulation by changing evaporation surfaces, which showed an evaporation rate of 2.6 kg·m^−2^·h^−1^ in practical applications.

The efficiency of the device is also affected when there are differences in temperature, humidity, light intensity, wind power, and incident light. Considering the different angles of natural sunlight and simulated light, Xu et al. [120] synthesized an inverted bowl-shaped graphene aerogel, which was used as a photothermal component in a solar evaporator. The structure of the device is shown in Figure 9b. The quasi-spherical light absorption surface is effectively improved compared to the 2D planar structure, which is the significance of the different structure design. Therefore, the evaporation amount on the bowl aerogel is higher than that on the normal flat aerogel. In addition, the shape of each cross-section of the inverted bowl-shaped graphene aerogel matches well with the trajectory of the sun, and the structural design is able to ensure a high evaporation rate output when the angle between the incident light and the horizontal plane is small (less than 20°), as shown in Figure 9c. The evaporation rate of the device is as high as 2.46 kg·m^−2^·h^−1^ at one solar irradiation under solar-driven airflow conditions.

Table 2 shows the comparison of the evaporation rate and efficiency parameters for different structures under one solar irradiation. Based on the above, a rational design of the structure of the solar interface evaporator is essential for improving evaporation and solar thermal conversion efficiency. Evaporators with the double-layer structure can greatly reduce heat transfer losses, which improvesf the overall utilization of solar energy. However, 2D interface solar evaporation systems need more capability and functionality to produce clean water [121,122]. Therefore, developing 3D and bionic-structured solar evaporation systems with high evaporation rates and energy conversion efficiencies is crucial. The unique design structure enables it to exhibit an excellent overall performance that provides new ideas for practical applications of solar interface evaporation devices.

## 5. Summary and Prospects

In conclusion, this review summarizes the development of solar-driven interfacial evaporators based on different kinds of photothermal materials and device structures. Notably, the advantages and disadvantages that exist according to the different types of photothermal materials are discussed, while their energy conversion mechanisms are elucidated. At the same time, the future direction of solar steam devices is discussed, with the aim of providing new ideas to alleviate the problem of freshwater resources.

Solar steam devices offer the possibility of efficient clean water generation because of the advantages of low energy consumption, scalability, and environmental friendliness. In recent years, as research has intensified, higher demands have been placed on the photothermal conversion rate of the devices. Improving the efficiency of solar-driven interfacial steam generators is based on three main aspects: optimizing light absorption, regulating water transport, and suppressing heat loss. Suitable photothermal materials usually have a laminar or porous microstructure that contributes to light capture. Excellent water transport layers generally exhibit tight honeycomb or regular pore structures, which are microchannels capable of promptly replenishing water lost to evaporation using superior capillary transport. Whichever material is used, it needs to maintain low thermal conductivity. The aim is to minimize heat loss from transport and convection processes to improve energy efficiency.

Additionally, most research on solar steam installations has focused on how to facilitate the evaporation process, ignoring the issue of salt accumulation during long-term operation. Therefore, the challenge remains to achieve large-scale applications of solar steam devices in seawater desalination. The design of steam structures and materials with high photothermal conversion performance is important for practical seawater purification applications where optically focused sunlight is not available. Challenges also include the construction of efficient salt deposition resistant structures that facilitate proper treatment of water delivery and blockage of water vapor escape channels after equipment fouling. In practical applications, the problem of salt deposition could not be ignored [123]. This may cause the solar steam generation device to fail in the long run, which hinders the stability of desalination performance. To solve the problem of sediment accumulation, one aspect can be to increase the porosity of the internal structure of the device. As the porosity increases, the interconnected water channels provide the possibility of high-water flux, which accelerates the rapid return and water supply of salt ions from high to low concentration seawater. Additionally, the design of the counter-diffusion and convection structures also contributes to the inhibition of salt accumulation. The main principle is that salinity differences drive convection and diffusion to return high concentrations of salt ions from the surface back to the water body in time. Nevertheless, resisting salt accumulation and maintaining good thermal management presents a complex coupling relationship [124,125]. Therefore, future strategies to minimize salt deposition and reduce heat losses are yet to be developed. Finally, surface modifications are used to make the light absorbing material resistant to acid, alkali, and oil. In particular, the absorbent layer is hydrophobically modified to prevent salt accumulation at the top, while the bottom is designed to be hydrophilic to facilitate rapid water transport. In this way, the solar steam device could operate for a long time in water sources with complex compositions that have stable performance output.

## Figures and Tables

**Table 1:** Comparison of evaporation rate and photothermal conversion efficiency of different materials under one solar irradiation.

	Materials	Evaporation Rate (kg·m^−2^·h^−1^)	Photothermal Conversion Efficiency	References
Carbon-based material	rGO/sodium alginate	1.60	83.00%	[59]
	CNTs/porous nickel mesh	2.13	94.3%	[62]
	Durable charred tofu	1.65	87.26%	[64]
Plasma metal	Gold nanostructured plasmon	2.70	79.30%	[75]
	Spherical silver nanoparticles	1.01	82.45%	[78]
	AgNPs/PDA	1.70	83.21%	[80]
Conjugated polymer	Conjugated polyphenylene diazole microspheres	2.96	90.30%	[81]
	Polypyrrole hydrogel	1.90	89.00%	[84]
	Novel porphyrin/aniline-based conjugated microporous polymer	1.31	86.3%	[87]
Inorganic semiconductor	MoS_2_	3.29	93.40%	[92]
	MnO_2_/CS	1.78	90.60%	[93]
	LaNiO_3_	2.30	83.00%	[94]

**Table 2:** The comparison of evaporation rates and efficiency parameters for different structures under one solar irradiation.

	Structures	Evaporation Rate (kg·m^−2^·h^−1^)	Evaporation Efficiency	References
Double-layer structure	Carbon particles/boxwood	1.00	65.00%	[100]
	Carbon particles/cellulose sponge	1.50	90.00%	[103]
Three-dimensional structure	A carbon-coated polyvinyl alcohol (PVA)/convection flower	3.31	166.10%	[111]
	rGO/cellulose sponge	4.35	178.80%	[105]
Biomimetic structure	Biomimetic mushroom	1.67	104.8%	[116]
	Carbonized sunflower	1.51	100.4%	[117]

## Data Availability

Not applicable.
